# A nomogram model to predict non-retrieval of short-term retrievable inferior vena cava filters

**DOI:** 10.3389/fcvm.2024.1393410

**Published:** 2024-12-06

**Authors:** Lihao Qin, Xiaocheng Gu, Caifang Ni, Kai Wang, Tongqing Xue, Zhongzhi Jia, Yun Wang

**Affiliations:** ^1^ Department of Interventional and Vascular Surgery, The Affiliated Changzhou Second People’s Hospital of Nanjing Medical University, Changzhou, China; ^2^ Department of Interventional Radiology, First Affiliated Hospital of Soochow University, Suzhou, China; ^3^ Department of Interventional Radiology, Huaian Hospital of Huai'an City (Huaian Cancer Hospital), Huai'an, China

**Keywords:** inferior vena cava, filter, retrieval, risk factor, OptEase, nomogram

## Abstract

**Objective:**

To develop and validate a nomogram for predicting non-retrieval of the short-term retrievable inferior vena cava (IVC) filters.

**Methods:**

In this study, univariate and multivariate logistic regression analyses were performed to identify predictive factors of short-term retrievable filter (Aegisy or OptEase) non-retrieval, and a nomogram was then established based on these factors. The nomogram was created based on data from a training cohort and validated based on data from a validation cohort. The predictive value of the nomogram was estimated using area under the curve (AUC) and calibration curve analysis (Hosmer-Lemeshow test).

**Results:**

A total of 1,321 patients who had undergone placement of short-term retrievable filters (Aegisy or OptEase) were included in the analysis. The overall retrieval rate was 68.7%. Age, proximal and distal deep vein thrombosis (DVT) vs. distal DVT, active cancer, history of long-term immobilization, VTE was detected in the intensive care unit, active/recurrent bleeding, IVC thrombosis, and history of venous thromboembolism were independent predictive risk factors for non-retrieval of filters. Interventional therapy for DVT, acute fracture, and interval of ≥14 days between filter placement and patient discharge were independent protective factors for non-retrieval of filters. The nomogram based on these factors demonstrated good ability to predict the non-retrieval of filters (training cohort AUC = 0.870; validation cohort AUC = 0.813.

**Conclusion:**

This nomogram demonstrated strong predictive accuracy and discrimination capability. This model may help clinicians identify patients who are not candidates for short-term retrievable filter placement and help clinicians make timely, individualized decisions in filter choice strategies.

## Introduction

**Figure 1:**
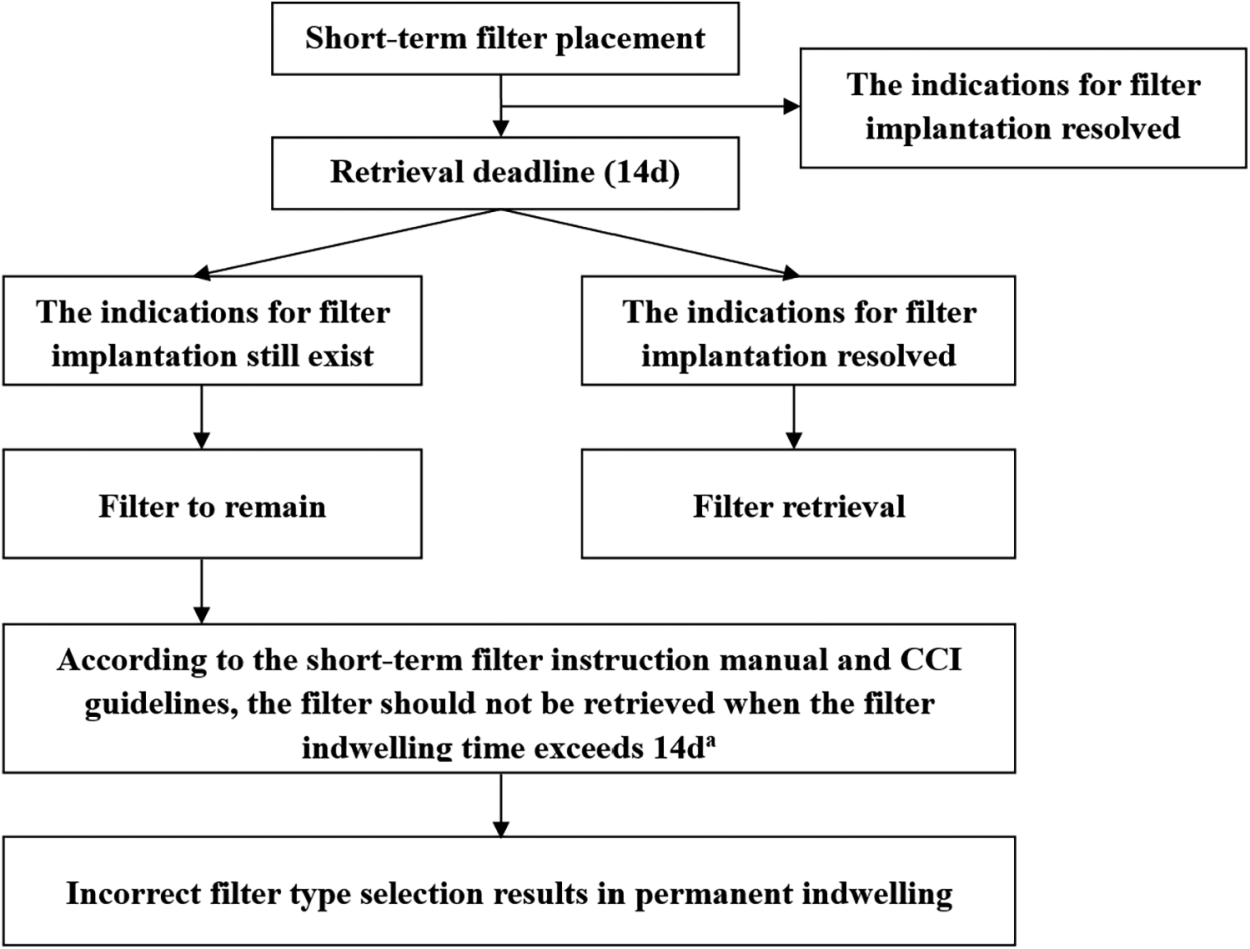
Short-term filter use patterns. CCI, Chinese College of Interventionalists. ^a^Vena cava endothelial hyperplasia and adhesion of the filters to the wall of the vena cava occurred when the short-term filter indwelling time exceeds 2 weeks (5).

**Figure 2:**
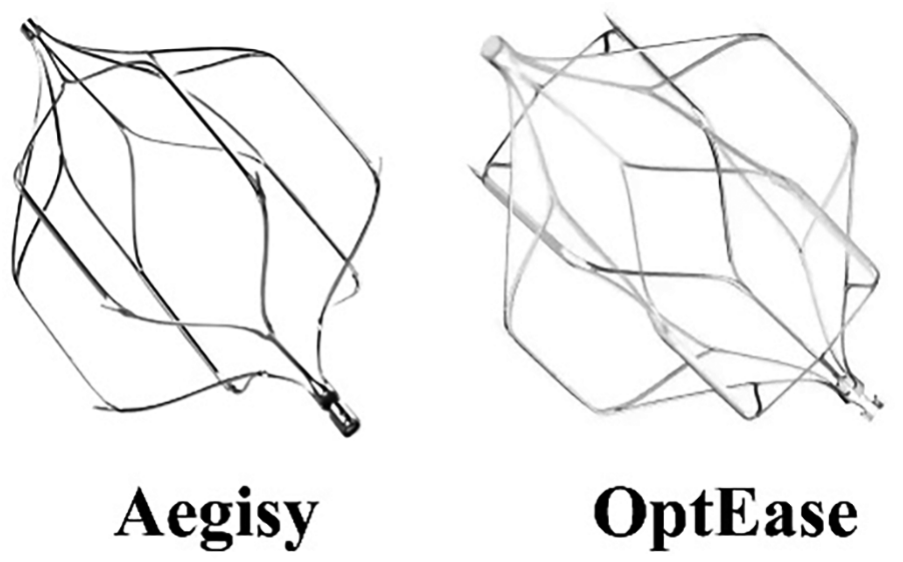
Images of Aegisy and OptEase filters. The short-term filters with a spindle-shaped appearance improve stability but also increase the contact area between the struts and the IVC wall, which can easily cause intimal hyperplasia to encase the struts.

Retrievable inferior vena cava (IVC) filters are devices that provide either temporary or permanent protection against the formation of pulmonary embolism (PE) (1). When a patient's clinical indication for PE protection no longer exists, the retrievable IVC filter can be retrieved to reduce the risk of potential long-term complications (2). There are two types of retrievable IVC filters commonly used in clinical practice, one is the “spindle” filter with retrieval window of 14 days, another is “umbrella” filter with no clear retrieval window (usually weeks to months) (3). The “spindle” filter is often not retrieved because the risk of PE is not eliminated at the end of retrieval window (Figure 1) (4). Long-term retention of the short-term filters may lead to complications (2). How to reasonably select different types of filters according to the situation of patients, to avoid the “spindle” filter non-retrieved due to the wrong choice strategy, is a clinical problem to be solved. The Aegisy (Lifetech Scientific, Shenzhen, China) and OptEase (Cordis, Santa Clara, California, USA) are the most commonly used short-term filters in China. They are similar in appearance and clinical characteristics (Figure 2).

The aim of this study was to determine the factors associated with non-retrieval of these short-term filters (Aegisy and OptEase) and to establish and validate a nomogram for predicting the probability of non-retrivability, so as to better guide physicians to choose the appropriate type of filter.

## Materials and methods

### Study design

The study population consisted of a training cohort and a validation cohort. The training cohort included patients with short-term filters (Aegisy or OptEase) filters placed at the affiliated changzhou second people's hospital of nanjing medical university (Hospital A) from January 2016 to May 2022. The validation cohort included patients with short-term filters (Aegisy or OptEase) filters placed at the first affiliated hospital of soochow university (Hospital B) from January 2016 to May 2022. All filter placements and venous thromboembolism (VTE) treatment were based on ESC guidelines for the diagnosis and management of acute PE and expert consensus guidelines from the Chinese College of Interventionalists (CCI) (4, 6, 7). The retention time of Aegisy and OptEase filters is normally ≤14 days.

The training and validation cohorts were subdivided into filter retrieval and non-retrieval groups. The nomogram was established using the training cohort and was validated using the validation cohort. All data were anonymized, and personal identifiers were completely deleted.

### Inclusion and exclusion criteria

Cases were included in the analysis if (1) the type of IVC filter placed was Aegisy or OptEase; (2) the filter placement was intended to be temporary. Cases were excluded from the final analysis if (1) the patients <18 years old; (2) patients with short life expectancy (not being requested to make a retrieval attempt); (3) the patient had died before filter retrieval attempt; (4) the patient had been lost to follow-up.

### Data collection

A list of potential predictors of filter non-retrieval was compiled based on clinical judgment and a search of the relevant literature (8–11). These predictors included (1) clinical characteristics of patients (sex, age, indication for filter placement, history of long-term immobilization, and department in which VTE was detected); (2) the occurrence of VTE events [clinical classification of deep vein thrombosis (DVT) or treatment of VTE]; and (3) the presence of concomitant conditions, including acute fracture (occurred in the previous 2 weeks), active cancer, acute cerebral hemorrhage/infarction, active/recent bleeding, iliac vein compression syndrome (IVCS), or IVC thrombosis.

### Statistical analysis

SPSS version 26.0 (IBM Corp, Armonk, NY, USA) was used for data analysis. Measurement data were expressed as M (P_25_, P_75_), with rank-sum tests used to compare groups. Count data were expressed as frequency (percentage), with differences between groups analyzed using chi-square tests or Fisher's exact probability method. *P* values less than 0.05 were considered statistically signiﬁcant.

### Construction of the nomogram

Univariate and multivariate logistic regression analyses were used to determine the independent predictors of non-retrieval for short-term filters filters in the training cohort. Variables signiﬁcantly related to the probability of non-retrieval in the univariate logistic regression analysis (*P* < 0.05) were subsequently included in the multivariate regression analysis. The nomogram was then constructed using *R* software (version 4.2.0) to visually score the individual probabilities for short-term filters filters non-retrieval.

### Performance of the nomogram

Receiver operating characteristic (ROC) curves and calibration curves were constructed to estimate the value of the nomogram in the training and validation cohorts. The discrimination performance of the nomogram was assessed using area under the curve (AUC). Calibration of the nomogram was evaluated using a calibration curve and a Hosmer-Lemeshow test [nonsignificance (*P* *>* 0.05) of the Hosmer-Lemeshow test indicates good agreement].

### Ethics approval

The study was conducted following the Declaration of Helsinki (as revised in 2013), and approved by the institutional review boards with waivers of informed consent. All data collection and analysis processes were performed in accordance with the institutional review board regulations.

## Results

### Patients

**Figure 3:**
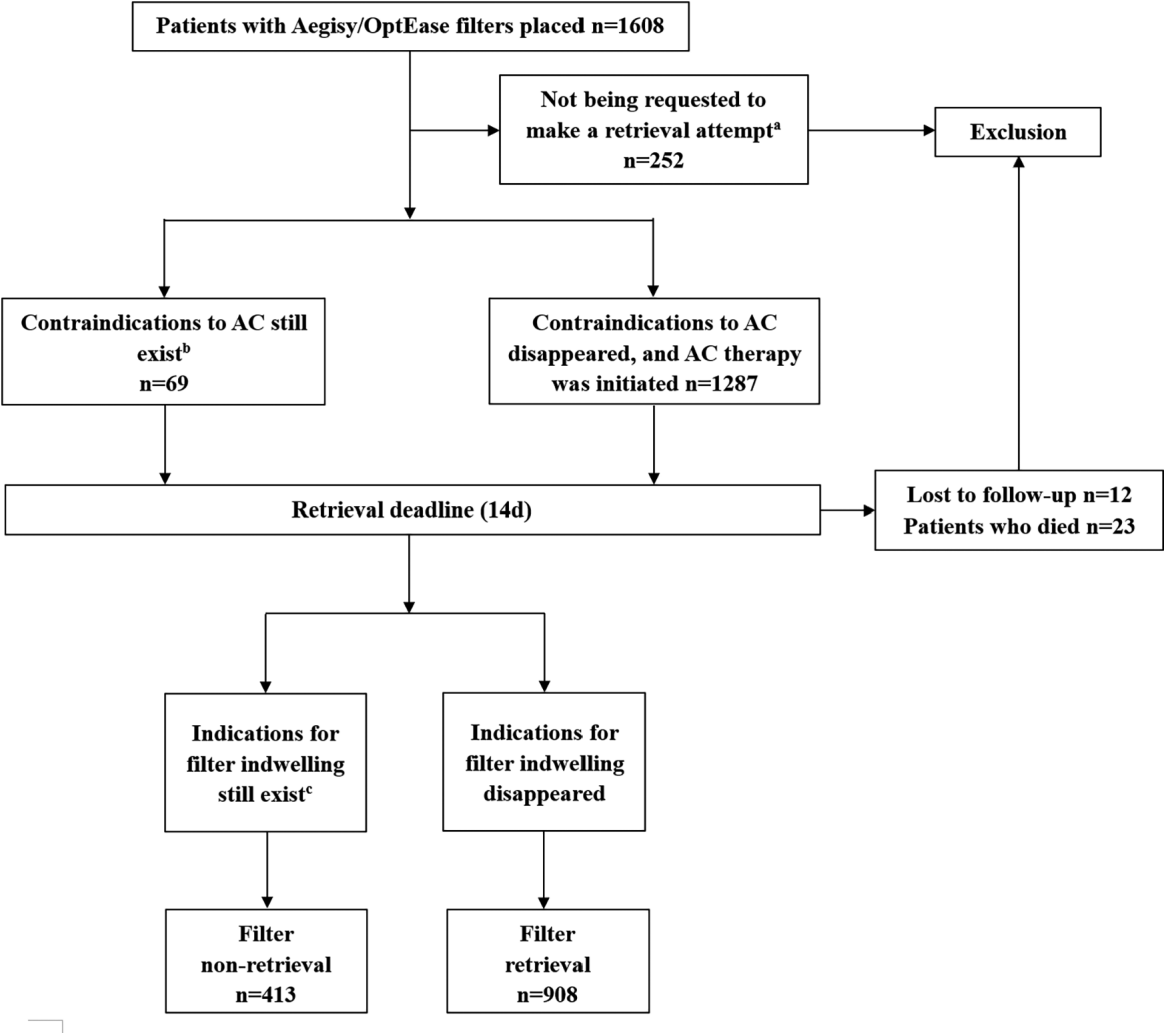
Patient selection flowchart. AC, anticoagulation. ^a^Patients with short life expectancy (discharge to hospice). ^b^Active/recent bleeding was not corrected. ^c^Propagation/progression VTE despite appropriate AC, inability to maintain adequate anticoagulation or complication of anticoagulation needs to be stopped.

During the study period, Short-term filters were placed in 1,608 patients. Of these patients, 287 were excluded from the analysis. The final study population therefore included 1,321 patients (826 patients in the training cohort, 495 patients in the validation cohort) (Figure 3). The overall filter retrieval rate was 68.7% (63.2% in the training cohort, 78.0% in the validation cohort). The reasons for the short-term filters filters non-retrieval were as follows: (1) propagation/progression of VTE despite appropriate anticoagulation (65.9%); (2) inability to maintain adequate anticoagulation, or complication of anticoagulation (21.3%); (3) contraindications to anticoagulation still exists (12.8%). Further information about filter placement and retrieval is shown in Table 1.

**Table 1:** Characteristics of filter placement and retrieval among study patients (*N* = 1,321).

Characteristic	No. of patients (%)
Indication for filter placement	
VTE with contraindication to AC	586 (44.4)
VTE with peri-operative needs to stop AC	459 (34.7)
Progression VTE despite appropriate AC	204 (15.4)
Recurrent VTE despite appropriate AC^a^	72 (5.5)
Type of filter	
Aegisy	1,169 (88.5)
OptEase	152 (11.5)
Filters retrieved^b^	908 (68.7)
Reason for non-retrieval of short-term filter	
Propagation/progression of VTE despite appropriate AC	272 (65.9)
Inability to maintain adequate AC, or complication of AC	88 (21.3)
Contraindications to AC still exists	53 (12.8)

AC, anticoagulation; VTE, venous thromboembolism.

^a^ During a single hospitalization, VTE recurred after treatment.

^b^ All filters were retrieved within the time specified by the manufacturer (14 d), and there was no failure of retrieved due to complications; Patients with thrombosis in the filter, the filter was all retrieved safely after thrombus aspiration.

### Independent predictors

Univariate analysis of the training cohort identified the following significant predictors of filter non-retrieval: patient age, DVT classification, interventional therapy for DVT, acute fracture, active cancer, IVCS, acute cerebral hemorrhage/infarction, history of long-term immobilization, DVT in ICU patient, active/recent bleeding, IVC thrombosis, history of VTE, and interval of ≥14 d between filter placement and patient discharge. Similar results were seen in the validation cohort (Table 2).

**Table 2:** Univariate logistic regression analysis for predictors of filter non-retrieval.

Variable	Training cohort, *n* (%)			Validation cohort, *n* (%)		
	Retrieval group (*n* = 522)	Non-retrieval group (*n* = 304)	*P* value	Retrieval group (*n* = 386)	Non-retrieval group (*n* = 109)	*P* value
Sex			0.642			0.789
Male	256 (49.0)	144 (47.4)		172 (44.6)	47 (43.1)	
Female	266 (51.0)	160 (52.6)		214 (55.4)	62 (56.9)	
Age, y	65 (56, 72)	73 (65, 80)	<0.001	63 (52, 72)	70 (62, 80)	<0.001
DVT clinical classification^a^			0.017			0.017
Proximal DVT	112 (21.5)	58 (19.1)		58 (15.0)	22 (20.2)	
Distal DVT	153 (29.3)	73 (24.0)		72 (18.7)	10 (9.2)	
Both proximal and distal DVT	158 (30.3)	125 (41.1)		256 (66.3)	76 (69.7)	
PE only^b^	99 (18.9)	48 (15.8)		0 (0)	1 (0.9)	
Interventional therapy for DVT			<0.001			<0.001
Yes	123 (23.6)	30 (9.9)		216 (56.0)	17 (15.6)	
No	399 (76.4)	274 (90.1)		170 (44.0)	92 (84.4)	
PE			0.202			0.666
Yes	148 (28.4)	99 (32.6)		179 (46.4)	48 (44.0)	
No	374 (71.6)	205 (67.4)		207 (53.6)	61 (56.0)	
Interventional therapy for PE			0.187			0.154
Yes	49 (33.1)	25 (25.3)		80 (44.7)	27 (56.2)	
No	99 (66.9)	74 (74.7)		99 (55.3)	21 (43.8)	
Acute fracture^c^			<0.001			0.026
Yes	177 (33.9)	50 (16.4)		116 (30.1)	21 (19.3)	
No	345 (66.1)	254 (83.6)		270 (69.9)	88 (80.7)	
Fracture site			0.090			0.628
Centrum	4 (2.3)	5 (10.0)		19 (16.4)	4 (19.0)	
Femur	90 (50.8)	19 (38.0)		50 (43.1)	11 (52.4)	
Long bones/joints of the extremities other than femur	35 (19.8)	10 (20.0)		18 (15.5)	1 (4.8)	
Multiple fractures	48 (27.1)	16 (32.0)		29 (25.0)	5 (23.8)	
Active cancer			<0.001			<0.001
Yes	30 (5.7)	96 (31.6)		68 (17.6)	46 (42.2)	
No	492 (94.3)	208 (68.4)		318 (82.4)	63 (57.8)	
IVCS^d^			<0.001			<0.001
Yes	72 (13.8)	16 (5.3)		140 (36.3)	20 (18.3)	
No	450 (86.2)	288 (94.7)		246 (63.7)	89 (81.7)	
Acute cerebral hemorrhage/infarction			0.001			<0.001
Yes	10 (1.9)	20 (6.6)		25 (6.5)	22 (20.2)	
No	512 (98.1)	284 (93.4)		361 (93.5)	87 (79.8)	
History of long-term immobilization^e^			<0.001			0.036
Yes	5 (1.0)	46 (15.1)		148 (38.3)	54 (49.5)	
No	517 (99.0)	258 (84.9)		238 (61.7)	55 (50.5)	
DVT in ICU patient			<0.001			<0.001
Yes	32 (6.1)	42 (13.8)		33 (8.5)	24 (22.0)	
No	490 (93.9)	262 (86.2)		353 (91.5)	85 (78.0)	
Active/recent bleeding^f^			<0.001			0.019
Yes	36 (6.9)	45 (14.8)		26 (6.7)	15 (13.8)	
No	486 (93.1)	259 (85.2)		360 (93.3)	94 (86.2)	
IVC thrombosis^g^			0.031			0.019
Yes	6 (1.1)	10 (3.3)		10 (2.6)	8 (7.3)	
No	516 (98.9)	294 (96.7)		376 (97.4)	101 (92.7)	
History of VTE^h^			0.027			<0.001
Yes	2 (0.4)	7 (2.3)		11 (2.8)	16 (14.7)	
No	520 (99.6)	297 (97.7)		375 (97.2)	93 (85.3)	
Interval of ≥14 d between filter placement and patient discharge^i^			<0.001			0.047
Yes	257 (49.2)	105 (34.5)		119 (30.8)	23 (21.1)	
No	265 (50.8)	199 (65.5)		267 (69.2)	86 (78.9)	

DVT, deep vein thrombosis; ICU, intensive care unit; IVC, inferior vena cava; IVCS, iliac vein compression syndrome; PE, pulmonary embolism; VTE, venous thromboembolism.

^a^ According to the location: Distal DVT refers to distal (or calf) DVT in the legs when it is found below the knee; Proximal DVT means a proximal (or iliofemoral) DVT in the legs above the knee.

^b^ PE only and contraindicated with anticoagulation.

^c^ Occurred in the previous 2 weeks.

^d^ Diagnosed via angiography before filter placement.

^e^ Long-term bed rest for more than 3 months.

^f^ VTE was detected while the patient is experiencing active/recurrent bleeding.

^g^ Thrombosis extending from the iliac vein to the inferior renal segment of the IVC, thrombus aspiration therapy was performed after filter placement over the IVC thrombus.

^h^ Occurring 3 or more months prior to filter placement.

^i^ 14 days was the end of filter retrieval window, the interval between filter placement and patient discharge ≥14 days, which meant that the patient completed filter placement and retrieval within one hospitalization.

In multivariate logistic regression analysis, age [odds ratio [OR] = 1.071; 95% confidence interval [CI]: 1.052–1.090], both proximal and distal DVT vs. distal DVT (OR = 1.763; 95% CI: 1.081–2.874), active cancer (OR = 12.112; 95% CI: 7.169–20.461), history of long-term immobilization (OR = 35.962; 95% CI: 12.503–103.441), DVT in ICU patient (OR = 3.807; 95% CI: 1.642–8.825), active/recent bleeding (OR = 4.879; 95% CI: 2.202–10.812), IVC thrombosis (OR = 13.116; 95% CI: 3.215–53.507), and history of VTE (OR = 12.534; 95% CI: 2.013–78.030) were identified as independent risk factors for non-retrieval of short-term filters filters. Interventional therapy for DVT (OR = 0.353; 95% CI: 0.202–0.619), acute fracture (OR = 0.461; 95% CI: 0.280–0.758), and interval of ≥14 d between filter placement and patient discharge (OR = 0.435; 95% CI: 0.286–0.660) were independent protective factors for non-retrieval of short-term filters filters (Table 3).

**Table 3:** Multivariable logistic regression analysis for predictors of filter non-retrieval.

Variable	OR (95% CI)	*P* value
Age, y	1.071 (1.052–1.090)	<0.001
DVT classification		0.020
Proximal DVT	1.148 (0.663–1.986)	0.623
Both proximal and distal DVT	1.763 (1.081–2.874)	0.023
Only PE	0.799 (0.453–1.410)	0.439
Interventional therapy for DVT (yes/no)	0.353 (0.202–0.619)	<0.001
Acute fracture (yes/no)	0.461 (0.280–0.758)	0.002
Active cancer (yes/no)	12.112 (7.169–20.461)	<0.001
History of long-term immobilization (yes/no)	35.962 (12.503–103.441)	<0.001
DVT in ICU patient (yes/no)	3.807 (1.642–8.825)	0.002
Active/recent bleeding (yes/no)	4.879 (2.202–10.812)	<0.001
IVC thrombosis (yes/no)	13.116 (3.215–53.507)	<0.001
History of VTE (yes/no)	12.534 (2.013–78.030)	0.007
Interval of ≥14 d between filter placement and patient discharge (yes/no)	0.435 (0.286–0.660)	<0.001

CI, conﬁdence interval; DVT, deep vein thrombosis; ICU, intensive care unit; IVC, inferior vena cava; OR, odds ratio; PE, pulmonary embolism; VTE, venous thromboembolism.

### Development and validation of a predictive nomogram

**Figure 4:**
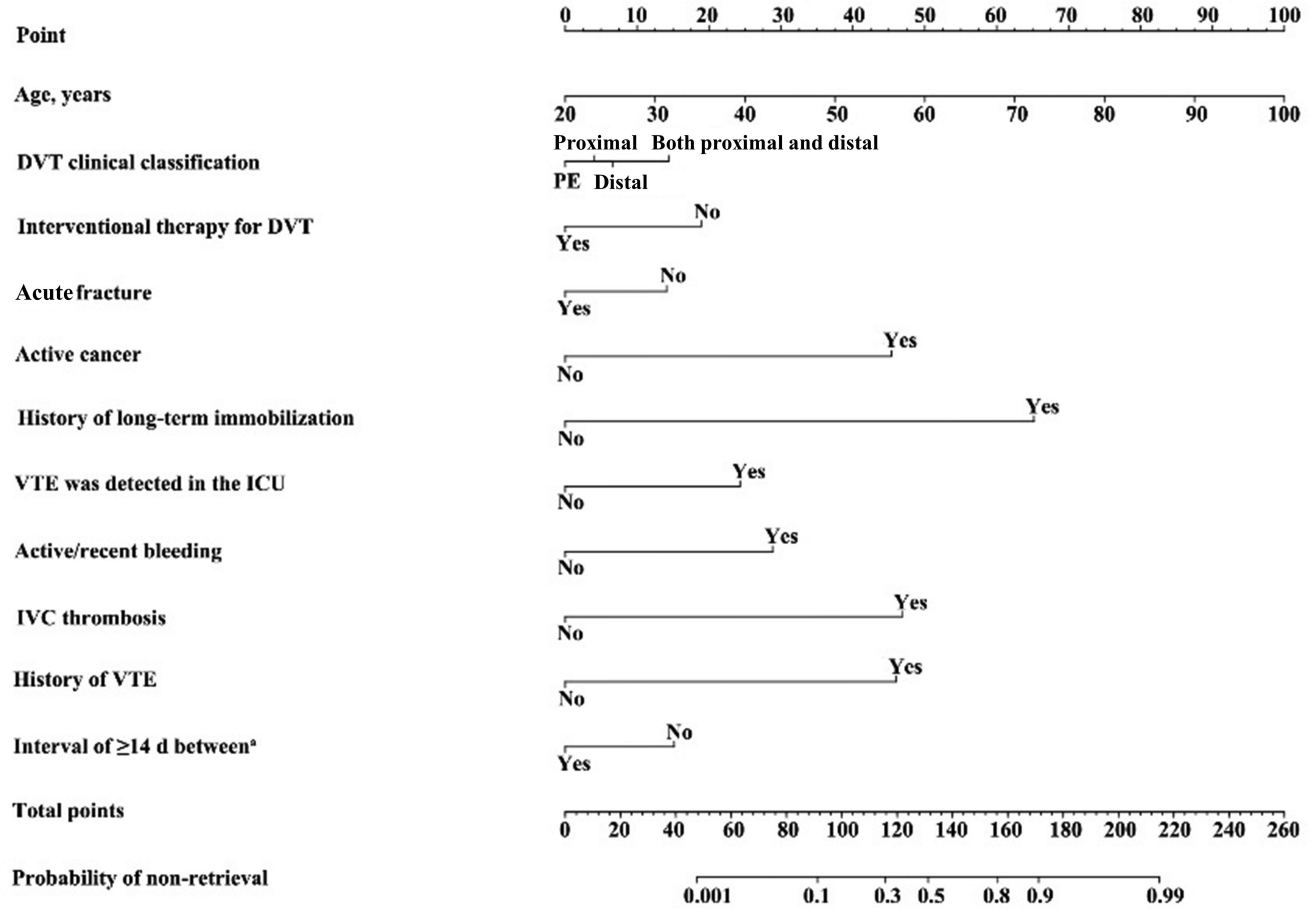
Predictive nomogram for risk of non-retrieval of Aegisy and OptEase filters. The points corresponding to each prediction variable were obtained. The sum of the points was then calculated as the total score, and the predicted risk corresponding to the total score was defined as the probability of filter non-retrieval. PE, pulmonary embolism only. ^a^Interval of ≥14 d between filter placement and patient discharge.

We used these 11 predictors of non-retrieval in a binary logistic regression analysis and transformed the results into a nomogram that could be used to predict the probabilities of filter non-retrieval (Figure 4). With this nomogram, the age of the patient was positioned on the corresponding variable axis; next, a vertical line was drawn to the “Points” axis to obtain the corresponding score (e.g., when age = 60, the corresponding score was 50). These steps were then repeated to obtain the scores for each variable, and all scores were summed to obtain the total score. This total score was identified on the “Total points” axis, and a vertical line was drawn to the “probabilities of non-retrieval” axis to determine the risk probabilities of non-retrieval.

**Figure 5:**
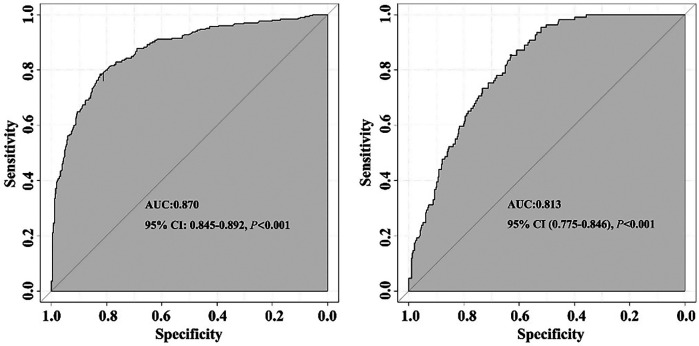
Receiver operating characteristic curves for validating the discrimination power of the nomogram prediction model. Training group. Validation group. AUC, area under the curve; CI, confidence interval.

**Figure 6:**
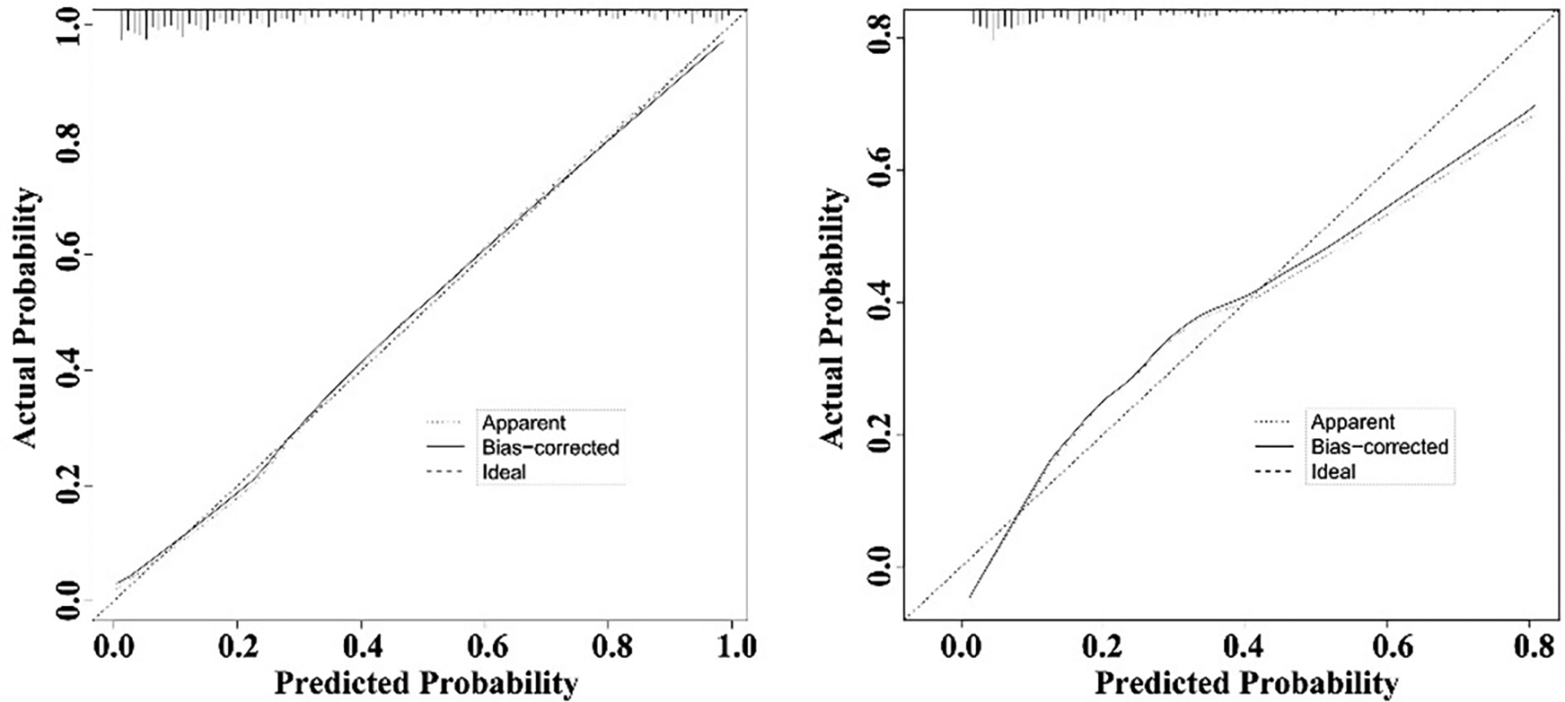
Calibration of the nomogram for the probability of non-retrieval of Aegisy and OptEase filters in training cohort and validation cohort. The Hosmer-Lemeshow test was not significant in the training cohort (*P* = 0.052) or in the validation cohort (*P* = 0.070).

The nomogram demonstrated a good ability to predict the non-retrieval of short-term filters. The AUCs were 0.870 (95% CI: 0.845–0.892) for the training cohort and 0.813 (95% CI: 0.775–0.846) for the validation cohort (Figure 5). The calibration curves of the nomogram showed good agreement between prediction and observation. The Hosmer-Lemeshow test was not significant in the training cohort (*P* = 0.052) or in the validation cohort (*P* = 0.070), which indicated a high reliability of the nomogram's predictive ability (Figure 6).

## Discussion

Short-term IVC filters are widely used in clinical practice, as they provide effective protection against PE and can be retrieval once they are no longer needed, and also has the advantage of not prone to tilt (12). In some cases, by the time the retrieval window for short-term filters arrives, the risk of PE and the contraindication to anticoagulation may still be present. These filters are often not retrieved because of the wrong strategy in choice of filter type, and long-term retention of the filters may lead to complications (13).

Previous research has focused on prediction model of non-retrieval filters to guide clinicians to decide whether to use temporary or permanent filters (14–18). This study was designed to identify risk factors for indications beyond the retrieval time window to guide clinicians to decide whether to use short-term or long-term filters. In this study, we found that short-term filters were retrieved in 68.7% of cases. The predictors of non-retrieval included age, DVT clinical classification, interventional therapy for DVT, acute fracture, active cancer, history of long-term immobilization, DVT in ICU patient, active/recent bleeding, IVC thrombosis, history of VTE, and interval of ≥14 days between filter placement and patient discharge. A nomogram we constructed based on these factors was found to offer a strong ability to predict non-retrieval of these filters.

Advanced age has previously been shown to increase the risk of bleeding during anticoagulation (14). With advancing age, venous valves degenerate, leading to valve incompetence, venous reflux, and increased venous pressure, all of which predispose patients to thrombus formation. Valve damage not only increases the risk of initial DVT but is also associated with post-thrombotic syndrome, resulting in persistent venous insufficiency and recurrent DVT (19–21). Moreover, due to the pharmacokinetic and pharmacodynamic changes in elderly patients, adjusting anticoagulant doses is complex. Even at standard therapeutic doses, elderly patients may experience bleeding complications, necessitating lower doses or more frequent monitoring of coagulation parameters, which can affect the effectiveness of anticoagulant therapy (21). Therefore, elderly patients with DVT present challenges in treatment and have a higher risk of recurrence. In our study, 191 patients were over 80 years old. Of these, 113 (59.2%) demonstrated acute DVT on re-examination, which resulted in the inability to retrieve the filter in a timely manner.

History of long-term immobilization is another known risk factor for VTE. In patients immobilized over a long period, blood flow is slowed and thrombosis is promoted (22). In this study, 253 patients had experienced long-term immobilization when VTE was detected; of these patients, 100 (39.5%) had progression of DVT during treatment, resulting in filters that could not be retrieved.

In this study, we found that fracture was an independent protective factor of the risk of filter non-retrieval. Although fracture itself is a risk factor for DVT, we found that in 1,321 patients, the filter retrieval rate in patients with fracture was higher than in patients without fracture (80.5% vs. 64.3%; *P* < 0.05). In our study, most patients with fractures were short-term contraindicated to anticoagulation or short-term discontinued anticoagulation due to surgery, so patients with fractures could be treated with timely anticoagulation after a brief observation. In addition, previous studies have shown that DVT in patients with fractures is usually found early thanks to standardization of early screening (23, 24). The key to treating DVT is early management (22). For patients with DVT due to fracture, most of the indications for filter placement have usually been eliminated within the deadline of filter retrieval, leading to high retrieval rates.

Active cancer is another risk factor for VTE (25). In addition, the risk of bleeding is substantially increased in patients with cancer because of the risk of thrombocytopenia after treatment, which has led to concerns regarding the use of anticoagulant drugs in these patients (26–28). In the current study, 240 (18.2%) patients had active cancer, filter placement was performed because of poor general condition, insufficient anticoagulation, and progression or recurrence of VTE. 183 (76.3%) of these were found to have DVT progression or large residual DVT at the time of proposed filter retrieval. Therefore, short-term filters should be used with caution in patients with active cancer, and clinicians must choose the type of filter based on the overall condition of the patient, life expectancy, and medical situation.

VTE detected in the ICU are usually in critically ill, and most of these patients have indwelling arteriovenous catheters, which increases the risk of VTE (29). In this study, DVT in ICU patient in 66 patients (50.4%) with contraindications to anticoagulation. They could not undergo filter retrieval because of an inability to maintain adequate anticoagulation and the progression of DVT.

History of VTE is another factor that must be considered in patients receiving short-term filters. In this study, 36 patients had a history of VTE and 23 patients (63.9%) were deemed non-retrievable due to progression of DVT. In such patients, it may be related to the presence of risk factors for thrombosis and an unreasonable anticoagulation regimen; the patient's compliance with this anticoagulation program may be poor. For patients with recurrent VTE, clinicians should therefore not only screen for refractory thromboembolism but should also adjust the anticoagulant treatment plan and strengthen follow-up. At the same time, short-term filters should be avoided as much as possible.

Patients with both proximal and distal DVT and IVC thrombosis are also at greater risk of DVT formation and thrombosis (22). In these patients, because of the large extent of the thrombosis, reflux can lead to decompensation, resulting in blood stasis, which can in turn aggravate the progression of DVT (22). Therefore, some of these patients who undergo short-term filters placement will continue to have a risk of PE that is not eliminated before the filter retrieval deadline, leading to indwelling of the filters.

Research has shown that interventional therapy for DVT can improve the rate of complete recanalization of the lumen, prevent venous valve adhesion, and reduce the incidence of valvular insufficiency and thrombosis recurrence (7). Therefore, DVT is cleared faster when interventional therapy is used. In this study, the use of interventional therapy was found to be a protective factor for non-retrieval of filters. Similarly, an interval of ≥14 days between filter placement and patient discharge was demonstrated to be a protective factor, perhaps because VTE in these patients was better prevented and treated in our department. Additionally, these patients were less likely to be lost to follow-up, since they did not need to readmission after discharge for retrieval after filter placement.

Overall, these results confirm the importance of filter choice when treating patients for VTE. The clinician can use this normogram to identify patients who are not candidates for short-term retrievable filter placement and conduct an individualized assessment of the patient receiving the retrievable filters placement to guide the clinician in selecting the appropriate type of filter and avoid associated complications caused by incorrect choice leading to long time indwelling of the filter. Our findings also suggest that clinicians should improve the supervision system of anticoagulation therapy, and that interventional thrombectomy should be performed when necessary to prevent the non-retrieval of filters because of the presence of residual.

This study had several limitations. First, although many risk factors can affect filter retrieval, our analysis included only the most important variables that can be readily assessed in clinical practice. Second, our model was only validated in the same region, and the performance of the model in regions with different management strategies is unknown. Finally, there was a difference in the retrieval rate between the training group and the validation group. The placement and retrieval of short-term filters in the two groups followed the same guidelines, and the difference in retrieval rate may be due to the difference in disease composition between the two groups.

In conclusion, this study demonstrated that age, DVT clinical classification, active cancer, history of long-term immobilization, VTE was detected in the ICU, active/recent bleeding, IVC thrombosis, history of VTE, interventional therapy for DVT, acute fracture, and interval of ≥14 days between filter placement and patient discharge were predictors of non-retrieval for short-term filters. The nomogram constructed in this study can provide clinicians with information to guide clinical decision-making and alter their strategy in choice of filter type to maximize the benefits for patients.

## Data Availability

The raw data supporting the conclusions of this article will be made available by the authors, without undue reservation.
